# A Multicenter Comparison of 1-yr Functional Outcomes and Programming Differences Between the Advanced Bionics Mid-Scala and SlimJ Electrode Arrays

**DOI:** 10.1097/MAO.0000000000004048

**Published:** 2023-10-27

**Authors:** Susan T. Eitutis, Deborah A. Vickers, Karen Tebbutt, Tisa Thomas, Dan Jiang, Anel de Klerk, Jennifer Clemesha, Mark Chung, Manohar L. Bance

**Affiliations:** ∗ Emmeline Centre, Cambridge University Hospitals NHS Foundation Trust; † Cambridge Hearing Group, Department of Clinical Neurosciences, Cambridge Biomedical Campus, University of Cambridge; ‡ Sound Laboratory, Cambridge Hearing Group, Clinical Neurosciences, University of Cambridge, Cambridge; § Guy's and St. Thomas' NHS Foundation Trust; ∥ Auditory Implant Department, Royal National ENT & Eastman Dental Hospitals, University College London Hospitals NHS Foundation Trust, London, United Kingdom

**Keywords:** Hearing performance, BKB, Cochlear implant, Hearing preservation, Programming, Stimulation levels

## Abstract

**Objective:**

To determine if there is a difference in hearing outcomes or stimulation levels between Advanced Bionics straight and precurved arrays.

**Study design:**

Retrospective chart review across three implant centers.

**Setting:**

Tertiary centers for cochlear and auditory brainstem implantation.

**Patients:**

One hundred fifteen pediatric and 205 adult cochlear implants (CIs) were reviewed. All patients were implanted under the National Institute for Health and Care Excellence 2009 guidelines with a HiRes Ultra SlimJ or Mid-Scala electrode array.

**Main outcome measures:**

Hearing preservation after implantation, as well as CI-only listening scores for Bamford-Kowal-Bench sentences were compared 1 year after implantation. Stimulation levels for threshold and comfort levels were also compared 1 year after implantation.

**Results:**

Hearing preservation was significantly better with the SlimJ compared with the Mid-Scala electrode array. Bamford-Kowal-Bench outcomes were not significantly different between the two arrays in any listening condition. Stimulation levels were not different between arrays but did vary across electrode contacts. At least one electrode was deactivated in 33% of implants but was more common for the SlimJ device.

**Conclusion:**

Modern straight and precurved arrays from Advanced Bionics did not differ in hearing performance or current requirements. Although hearing preservation was possible with both devices, the SlimJ array would still be the preferred electrode in cases where hearing preservation was a priority. Unfortunately, the SlimJ device was also prone to poor sound perception on basal electrodes. Further investigation is needed to determine if deactivated electrodes are associated with electrode position/migration, and if programming changes are needed to optimize the use of these high-frequency channels.

## INTRODUCTION

A cochlear implant (CI) is an implantable hearing device that provides sound through electrical stimulation. The design of a CI electrode array influences where the device is located within the cochlea and can impact the amount of stimulation (current) required to reach audibility (1–3), the risk of cochlear damage (4), and hearing performance after implantation (5,6).

Precurved arrays are designed to curl toward the modiolus in an effort to keep electrical stimulation close to the remaining auditory-neural tissues (7–9). This perimodiolar (PM) placement helps minimize the amount of current needed to activate the auditory nerve (1,2,8,10) and potentially offers more focused stimulation (11). Given the relationship between current, impedances, and compliance, minimizing current may be advantageous for maintaining stimulation rate, sound quality, and battery life (10,12). In addition, electrodes positioned closer to the modiolus have been correlated with better word recognition (5,6), improved electrode discrimination (13,14), and reduced listening effort (13). However, cochlear tissue damage may be more prevalent with PM designs because of their higher risk of tip fold-over and scalar translocation (4,15–18).

In contrast, lateral wall (LW) arrays are “straight” electrodes designed to sit away from the modiolus. They are farther from the neural tissue and may require more current for auditory stimulation (1,19,20). However, the simpler array design and insertion process helps reduce the risk of cochlear damage and the incidence of translocation (21,22). Minimizing cochlear damage has implications for hearing preservation (HP), which has historically been better for LW designs (23,24), and may be beneficial in challenging listening environments when using electroacoustic stimulation (EAS) (25–27).

Conventional evidence favors PM arrays for “traditional” more severely deafened CI candidates and LW arrays for those with more hearing (28). However, with the advent of soft surgery techniques and less traumatic electrode designs, HP rates have improved for PM arrays (22,29). Implant centers must now weigh the risks and benefits of modern array designs to determine if the benefits associated with modiolar proximity are still relevant (5,6,13,14).

With this retrospective analysis of our implanted population, we compared the Advanced Bionics (Advanced Bionics, LLC, Valencia, CA) SlimJ and Mid-Scala electrodes to determine if conventional evidence holds true for these devices. Both arrays have demonstrated structural preservation in temporal bones, which is important for HP (30). However, there are still design differences that may be important to electrode selection. The SlimJ is an LW electrode with a narrower diameter than the Mid-Scala device (31), whereas the Mid-Scala is a precurved electrode that does not hug the modiolus closely. Instead, it floats in the “middle” of the scala tympani (22) to offer a flexible free-fit with minimal damage (32–34). To date, studies have compared histologic and radiological outcomes between the Mid-Scala and SlimJ electrodes (30), but few studies have compared patient outcomes (18).

For modern straight and precurved Advanced Bionics arrays, we aimed to investigate 1) if there was a difference in HP, 2) if hearing outcomes supported conventional choices for PM versus LW arrays, and 3) if the Mid-Scala device required lower current levels than the SlimJ device because of its position closer to the modiolus. We hypothesized that HP would be superior with the LW electrode, and that electrical hearing would be better with the precurved electrode.

## METHODS

A multicenter retrospective chart review across three UK CI centers was conducted for 63 pediatric patients (115 implants) and 204 adult patients (205 implants) implanted between January 2016 and November 2018 (Table 1). This was registered as an audit, and no formal ethics approval was required at our centers (PRN:7724).

**TABLE 1:** Summary of adult and pediatric implants reviewed for the multicenter comparison between Mid-Scala and SlimJ electrode arrays

	Adult Patients		Pediatric Patients	
	SlimJ	Mid-Scala	SlimJ	Mid-Scala
No. implants reviewed	102	103 (1-B)	46 (21-B, 4-U)	69 (31-B, 7-U)
Prelingual deaf/hearing loss*^a^*	19	29	30	32
BSL or nonverbal*^b^*	5	7		
Number V1 faults*^c^*	4	12	2	11
No 1-yer appointment*^c^*	5 DNA/CNA, 1 deceased	6 DNA/CNA		1 CNA, 2 (1 deceased—B)

*^a^* Patients with a hearing loss identified at or before the age of 3 years were defined as prelingual hearing loss.

*^b^* Those who did not use spoken language as primary mode of communication were excluded from all comparisons.

*^c^* Patients identified with V1 faults or who did not attend their 1-year appointment were excluded from functional outcome and programming comparisons.

B indicates bilateral cochlear implants; BSL, British Sign Language; CNA, could not attend; DNA, did not attend; U, unilateral cochlear implant.

All patients were implanted with Advanced Bionics HiRes Ultra Mid-Scala or SlimJ electrodes under the National Institute for Health and Care Excellence 2009 guidelines (35). Hearing thresholds before and after CI surgery, etiology, and age of onset for hearing loss were collected for each patient.

Low-frequency preoperative hearing thresholds at 250 and 500 Hz were used to differentiate between more severely deafened patients and potential EAS candidates. Thresholds for 125 Hz were not routinely measured, so could not be included in comparisons of hearing thresholds or HP.

The distribution of preoperative hearing at 250 and 500 Hz was normal for SlimJ and Mid-Scala recipients (*p* > 0.05, Kolmorogov-Smirnoff; Supplemental Fig. 1, http://links.lww.com/MAO/B771). There was no significant difference between devices for preoperative hearing at 250 (average, 79.0 dB HL [SlimJ], 82.5 dB HL [Mid-Scala]; *t* = −1.06, *p* = 0.29) or 500 Hz (average, 86.9 dB HL [SlimJ], 94.6 dB HL [Mid-Scala]; *t* = −0.79, *p* = 0.43), indicating that preoperative hearing did not bias electrode selection.

### Hearing Preservation

Degree of HP (% HP) and HP classification at low frequencies was calculated using a modified HEARRING group formula (23,36,37), by including only 250- to 500-Hz thresholds. To ensure no audiograms were missed, hearing tests completed up to 6 months after implantation were collected; however, most were completed within 3 months of surgery (n = 127 of 131).

### Hearing Outcomes

Bamford-Kowal-Bench (BKB) sentence (% words correct) scores were collected for CI-only listening conditions 1 year after implantation (12 ± 4 mo) for adult recipients. Scores were collected for BKB sentences when listening to a male speaker in quiet, female speaker in quiet, male speaker in noise, and female speaker in noise conditions. All speech in noise testing used pink noise at +10 dB signal-to-noise ratio.

To check if more severely deafened CI candidates should receive the precurved array and those with better hearing the LW array, BKB scores were compared based on preoperative hearing for 250 and 500 Hz separately. Patients were grouped into the following ranges for each frequency: (*a*) hearing likely to benefit from EAS (≤70 dB HL) and (*b*) hearing unlikely to benefit from EAS (>70 dB HL). Because of the limited use of combined stimulation, comparisons for active EAS components could not be made.

### Programming Details

Threshold level, comfort level, pulse width, and number of deactivated electrodes were collected 1 year after implantation. Threshold and comfort levels were presented in charge units (cu), as listed in the SoundWave software (Advanced Bionics, LLC, Valencia, CA). This allowed for direct comparison of clinical MAPs, as cu already incorporates pulse width [cu = (microAmps × pulse width)/79]. Levels were also converted to charge [nC/phase = (cu × 79)/1000] to enable comparison with other manufacturers. Sound-field aided thresholds 1 year after implantation confirmed that sound detection was within the clinically recommended range (20–30 dB HL (38,39)) for most patients (Supplementary Fig. 2, http://links.lww.com/MAO/B772), indicating acceptable threshold levels in most cases. Thresholds were measured at centers 1 and 2, but left at default (10% of comfort levels) for most adults at center 3. Threshold levels were also set to default for most pediatric patients at centers 1 and 2.

### Exclusions

Patients who did not use spoken language as their primary mode of communication (5 SlimJ, 7 Mid-Scala) were excluded from all comparison. Patients were also removed if they did not attend their 1-year appointment (6 SlimJ, 9 Mid-Scala), or showed signs of V1 failure (40,41) (6 SlimJ, 23 Mid-Scala; Table 1).

### Statistics

All tests were completed using SPSS 26 statistics package (SPSS Inc., Chicago, IL) (42). Normality was checked using the Kolmorogov-Smirnoff test. Arrays were compared to check for differences in preoperative hearing (independent *t* test), HP (Mann-Whitney *U* and Kruskal-Wallis tests), overall BKB outcomes (Mann-Whitney *U* test), and variance in BKB outcomes (Levene test). In addition, interactions were checked for BKB outcome using univariate analysis of variance (ANOVA), with factors electrode (SlimJ versus Mid-Scala) and hearing threshold (≤70 versus >70 dB HL) for 250 and 500 Hz separately. Distribution of threshold and comfort levels were tested separately using repeated-measures ANOVA, with factors channel (electrodes 1–15, electrode 16 excluded) and implant center (1 or 2, 3 excluded). Threshold and comfort levels were significant when tested against the Mauchley test of sphericity, therefore, the Greenhouse-Geisser correction was used. Finally, the number of deactivated channels was analyzed with the *χ*^2^ test.

## RESULTS

### Do Arrays Differ in HP?

**FIG. 1:**
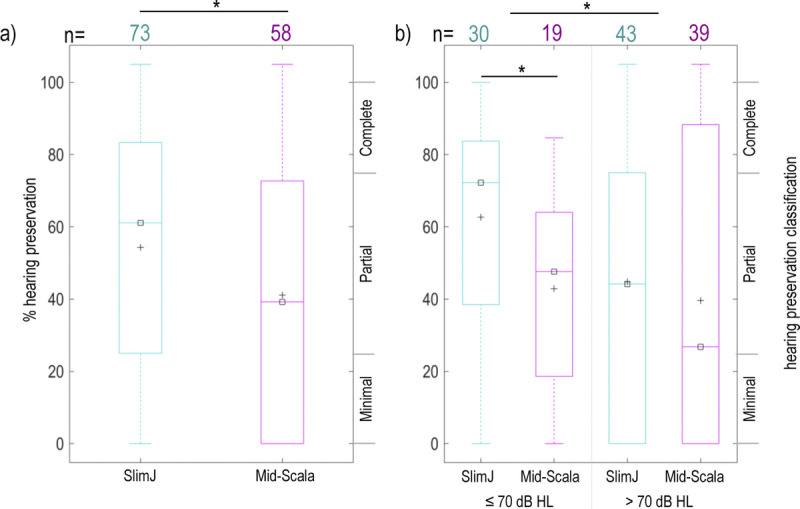
Percentage (%) hearing preservation after cochlear implant surgery. *A*, Hearing preservation shown by electrode, regardless of preoperative hearing thresholds. *B*, Hearing preservation by electrode based on preoperative hearing thresholds for 250 Hz. The number of patients is listed for SlimJ (teal) and Mid-Scala (pink) electrodes, along with the mean (+) and median (☐) for each measurement (*p < 0.05, Mann-Whitney *U*).

Postoperative hearing was measured for 131 ears (73 SlimJ, 58 Mid-Scala). Degree of HP was not normally distributed (Kolmogorov-Smirnoff = 0.13, *p* < 0.001). Overall, degree of HP was better for the SlimJ device (average HP: 54% SlimJ, 41% Mid-Scala; Mann-Whitney *U* = 1,675, *p* = 0.04; Fig. 1), with no effect of center (Kruskal-Wallis = 1.04, *p* = 0.59).

The degree of HP was better for EAS candidates (average HP: 57%) compared with non-EAS candidates (average HP: 43%), with a significant effect of electrode (Mann-Whitney *U* = 1,548, *p* = 0.028). The electrode effect showed better HP with the SlimJ device for EAS candidates (average HP: 66% SlimJ, 42% Mid-Scala; Mann-Whitney *U* = 151.5, *p* = 0.006), but no significant difference for non-EAS candidates (average HP: 46% SlimJ, 40% Mid-Scala; Mann-Whitney *U* = 760, *p* = 0.462). Despite at least some HP in most cases, EAS was active for only seven adult and eight pediatric implants 1 year after implantation.

### Do Hearing Outcomes Differ Between Arrays?

**FIG. 2:**
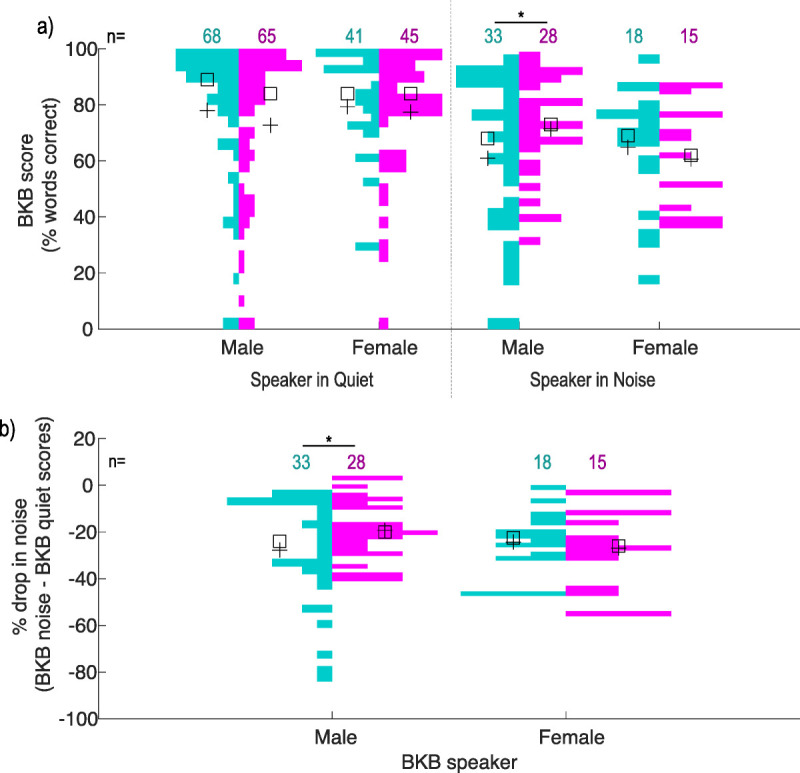
BKB results 12 months after activation for adult patients only. *A*, Results shown for each test condition. *B*, Difference in BKB scores when comparing noise and quiet conditions for the same speaker. % drop in noise refers to the percentage point difference between the noise and quiet condition. The data are plotted as back-to-back histograms, where the size of the colored bars reflects the number of individuals with BKB scores in that % range. The number of measurements in each listening condition is listed for SlimJ (teal) and Mid-Scala (pink) electrodes, with the mean (+) and median (☐) shown for each group. Overall outcomes remained similar between both electrode arrays in all conditions tested; however, a greater variation was measured when listening to a male speaker in noise for the SlimJ array (*p < 0.006, Levene test). BKB indicates Bamford-Kowal-Bench.

BKB outcomes were skewed for both arrays in all test conditions (Kolmorogov-Smirnoff, *p* ≤ 0.007) and were limited by ceiling effects (Fig. 2).

Overall, speech perception was not different between the arrays in any BKB test condition (average: male speaker in quiet [78% SlimJ, 73% Mid-Scala; Mann-Whitney *U* = 1960.0, *p* = 0.26], female speaker in quiet [79% SlimJ, 77% Mid-Scala; Mann-Whitney *U* = 892.5, *p* = 0.80], male speaker in noise [61% SlimJ, 71% Mid-Scala; Mann-Whitney *U* = 545.5, *p* = 0.23], female speaker in noise [65% SlimJ, 59% Mid-Scala; Mann-Whitney *U* = 118.0, *p* = 0.56]). However, there was a greater spread in performance with a male speaker in noise for the SlimJ device (*F* = 7.965, *p* = 0.006; Levene test; Fig. 2A). The spread of performance was not significantly different for tests in quiet and could not be compared for the female speaker in noise because of small sample sizes.

To check if one electrode was better for speech in noise, we examined how performance reduces when listening in noise compared with when listening in quiet. The average decrease in performance was similar between the two arrays for both speakers (average percentage point decrease: male [27 SlimJ, 20 Mid-Scala; Mann-Whitney *U* = 534, *p* = 0.297], female [24 SlimJ, 27 Mid-Scala; Mann-Whitney *U* = 123.5, *p* = 0.682]). However, there was greater variability in outcomes for the male speaker with the SlimJ electrode (Levene test, *F* = 8.396, *p* = 0.005; Fig. 2B).

#### Hearing Performance Versus Preoperative Thresholds

**FIG. 3:**
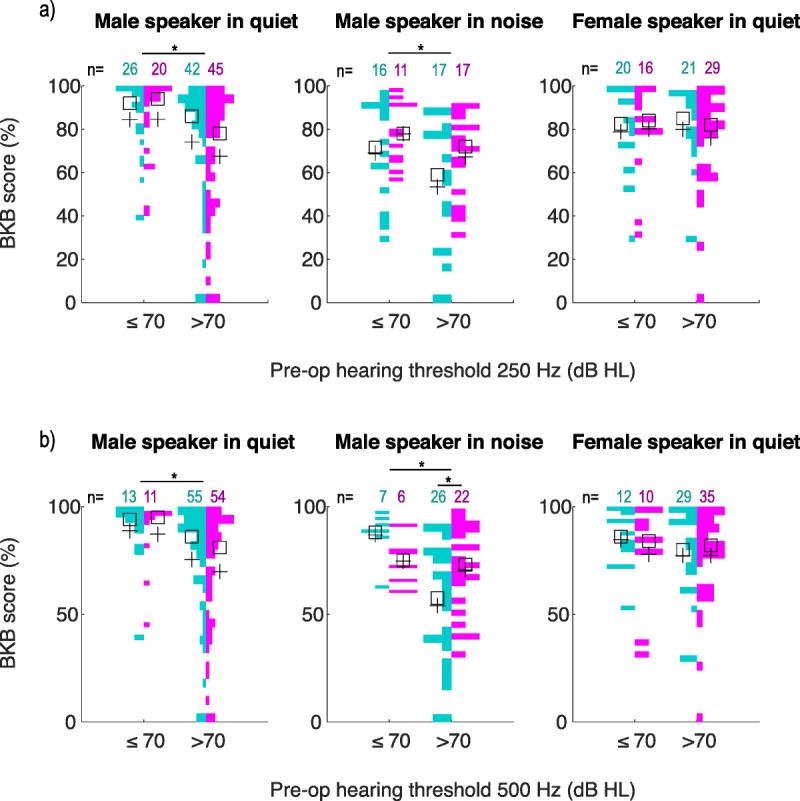
BKB outcomes 9 to 12 months after activation for EAS candidates (preoperative hearing ≤70 dB HL) and non-EAS candidates (preoperative hearing >70 dB HL) based on preoperative hearing thresholds. *A*, BKB scores based on 250-Hz preoperative thresholds. *B*, BKB scores based on 500-Hz preoperative thresholds. The data are plotted as back-to-back histograms, where the size of the colored bars reflects the number of individuals with BKB scores in that % range. The number of measurements in each listening condition is listed for SlimJ (teal) and Mid-Scala (pink) electrodes, with the mean (+) and median (☐) shown for each group. Results for female speaker in noise are not shown as the sample size was too small. Differences were only detected in BKB male speaker conditions between EAS candidates (preoperative hearing ≤70 dB HL) and non-EAS candidates (preoperative hearing >70 dB HL), regardless of electrode type. For 500 Hz, BKB scores when listening to a male speaker in noise were lower for non-EAS candidates with the SlimJ compared with the Mid-Scala electrode (*p < 0.05, ANOVA). ANOVA indicates analysis of variance; BKB, Bamford-Kowal-Benchl; EAS, electroacoustic stimulation.

To test if LW arrays should be the preferred choice for EAS candidates (i.e., preoperative low-frequency hearing better than 70 dB HL), preoperative hearing was compared separately for 250 and 500 Hz. For each frequency, BKB results were compared between EAS candidates (≤70 dB HL) and non-EAS candidates (>70 dB HL; Fig. 3).

Regardless of electrode, patients with preoperative hearing ≤70 dB HL had better BKB scores when listening to a male speaker in quiet and in noise. This held true for 250 and 500 Hz (average: ≤70 versus >70 dB HL for 250 Hz in quiet [84% versus 71%; ANOVA, *F*_1,129_ = 8.15, *p* = 0.005], 250 Hz in noise [74% versus 60%; ANOVA, *F*_1,57_ = 4.48, *p* = 0.04], 500 Hz in quiet [88% versus 73%; ANOVA, *F*_1,129_ = 6.64, *p* = 0.01], 500 Hz in noise [81% versus 62%; ANOVA, *F*_1,57_ = 6.72, *p* = 0.01]).

There was also an interaction when listening to a male speaker in noise between electrode and frequency (ANOVA, *F*_1,57_ = 4.06, *p* = 0.049), where performance was worse for SlimJ recipients, but only when preoperative hearing was >70 dB HL at 500 Hz (average: 54% SlimJ, 71% Mid-Scala). A similar trend was observed for 250 Hz but was not significant (average: 53% SlimJ, 67% Mid-Scala; ANOVA, *F*_1,57_ = 3.32, *p* = 0.07). However, this interaction is likely attributed to six SlimJ recipients who experienced significant difficulty when listening in noise (Fig. 2B).

When listening to a female speaker in quiet, BKB scores were similar regardless of preoperative hearing and electrode (ANOVA, *p* > 0.05). Unfortunately, because of limited sample size, the female speaker in noise condition could not be analyzed. Regardless of electrode, patients with better preoperative hearing at 250 or 500 Hz tended to have higher BKB scores only when listening to the male speaker.

### Does the Mid-Scala Device Require Lower Stimulation Levels?

One-year programming MAPs were collected for 131 SlimJ and 133 Mid-Scala implants. Deactivated electrodes were more common for the SlimJ device, with 60 (46%) SlimJ and 42 (32%) Mid-Scala devices having at least one electrode turned off (*χ*^2^ = 4.69, N = 265, *p* = 0.03). This trend was common across all centers, as well as for adult and pediatric recipients (Supplementary Table 1, http://links.lww.com/MAO/B774). Basal electrodes were most frequently deactivated for both arrays. The most common reason was inadequate sound perception, including poor sound detection or loudness growth (reasons in Supplementary Table 2, http://links.lww.com/MAO/B775).

#### Current Levels

**FIG. 4:**
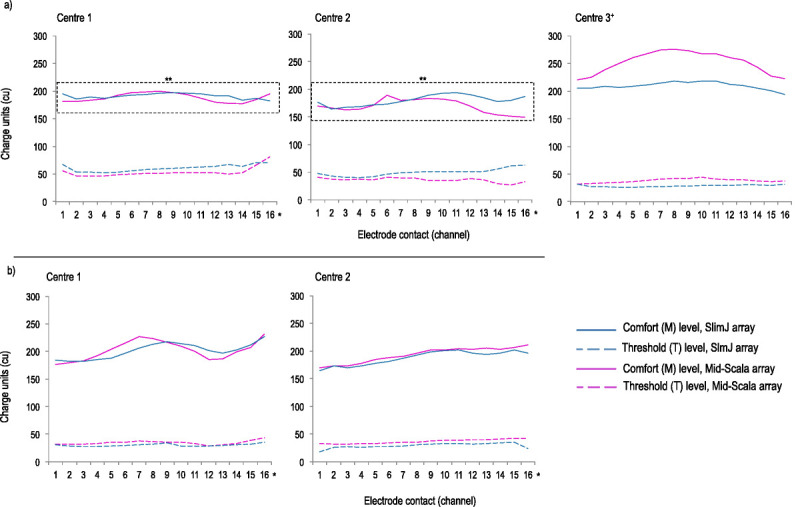
Average threshold (T) and comfort (M) levels at each site for adult patients (*A*) and pediatric patients (*B*) who attended their 9- to 12-month programming appointments. All levels are shown as charge units (cu) obtained from the SoundWave programming software. *T and M levels varied across electrode contacts (1–15) for adult and pediatric patients (*F* > 7, *p* < 0.001, ANOVA). **M levels in adults only showed an interaction between channel and electrode array (SlimJ/MS). ^+^Center 3 was not included in ANOVA but shows higher M levels. ANOVA indicates analysis of variance; MS, Mid-Scala.

Average threshold and comfort levels are shown separately for each center because of differences in programming (Fig. 4; Supplementary Figure 3, http://links.lww.com/MAO/B773). For adults (Fig. 4A), there was no significant main effect of electrode or center for threshold or comfort level. There was, however, a significant effect for electrode channel (electrodes 1–15) on threshold (*F*_2.8,253.5_ = 7.04, *p* < 0.001), and comfort level (*F*_3.3,295.0_ = 8.8, *p* < 0.001). Similarly, there was an interaction for comfort level between channel and array (*F*_3.3,295.0_ = 4.9, *p* = 0.001), where arrays had different comfort levels, but only for basal channels. However, to address our question, overall current requirements were similar for both devices.

Comfort levels for center 3 appeared elevated compared with centers 1 and 2 (Fig. 4A). However, center 3 was not included in the ANOVA because of using default (10% of comfort levels) rather than measured thresholds.

For pediatric patients, there was no significant main effect of center or electrode for threshold or comfort level. There was, however, a significant effect for electrode channel on threshold (*F*_3.6,284_ = 11.6, *p* < 0.001) and comfort level (*F*_3.7,290_ = 29.9, *p* < 0.001; Fig. 4B). No interaction between array and channel was observed, although this may be related to reliance on objective measures rather than behavioral-based programming approaches.

## DISCUSSION

### Do Arrays Differ in HP?

HP was better with the SlimJ compared with the Mid-Scala array. This aligns with previous studies showing better HP for LW than PM electrodes (23,43,44). Although HP may be better with LW arrays, it is still often possible to preserve hearing with precurves designs (45,46). Recent studies have even shown similar rates of HP between PM and LW arrays (22,47). However, HP is multifactorial, with factors beyond electrode design under the surgeons' control.

Preoperative hearing also impacted HP, where those with better hearing had better HP. Here, the best HP results were for SlimJ recipients whose preoperative hearing fell within EAS candidacy. This relationship between preoperative hearing and HP has previously been reported (44,48). However, not all studies have supported this correlation (49).

Despite the clinical importance, there is limited consistency when reporting HP. HP must be interpreted cautiously, particularly when comparing across different measurement approaches. “Functional hearing” preservation will vary depending on the tolerances used for threshold change or percentage change measurements, and by which frequencies were included in the calculations (37).

### Do Hearing Outcomes Differ Between Arrays?

We found no significant difference in average BKB performance between arrays. There was no clear benefit for Mid-Scala recipients with more “traditional” preoperative hearing thresholds, nor was there a benefit for EAS candidates implanted with the SlimJ device. These data suggest that modern Advanced Bionics arrays do not differ in aided hearing outcomes based on preoperative hearing.

This aligns with recent studies reporting no significant difference on sentence or disyllabic tests between modern precurved and straight arrays (13,50–55). However, some studies have reported better performance for PM arrays on electrode discrimination (13), listening effort (13), and consonant nucleus consonant tests (47,51,55,56). In the largest review to date, PM arrays outperformed straight arrays on AzBio sentences only after considering additional factors (47). Although we did find an effect for Mid-Scala recipients with poorer preoperative hearing, this was limited only to the distribution of BKB outcomes when listening to a male speaker in noise. This could point to a potential benefit of the Mid-Scala array in challenging listening environments for more traditional candidates. However, it may also be skewed by six SlimJ recipients who had disproportionately more difficulty (drop of >40% points) in noise compared with quiet listening conditions. This degree of difficulty was not observed for any Mid-Scala recipients, nor could it be explained by prelingual/postlingual deafness, etiology, age at implant, sound-field aided threshold, number of deactivated electrodes, or threshold/comfort levels.

When comparing outcomes between potential EAS candidates, the similarity in performance between arrays may be related to the limited use of combined stimulation. Historically, benefit from HP has been associated with EAS use (21,26,27,57). Unfortunately, despite successful HP, only 15 of our implants had active EAS components 1 year after implantation. Because most of these were pediatric recipients with no BKB testing, we were unable to compare hearing outcomes for this group.

Given similarities in performance between modern straight and precurved devices, electrode selection may be driven by factors such as cochlear anatomy or etiology rather than hearing thresholds. We are, however, mindful that BKB sentences may not be sensitive enough to detect potential benefits of HP or modiolar proximity. Single word or nonspeech tests may better highlight performance differences between array designs. In addition, investigation into the limited use of EAS is needed to determine what barriers led to such poor uptake of combined stimulation.

### Does the Mid-Scala Device Require Lower Stimulation Levels?

There was clear variation in current requirements across electrode channels, but no difference between arrays for overall current levels. This could be a reflection of the Mid-Scala array placement, where cochlear position ranges from entirely midscalar to mostly LW depending on cochlear size (20,34,58). However, these results are consistent with recent studies showing similar charge requirements between modern PM and LW arrays (51,56).

Current levels by channel reflect previously published data about scalar location (Fig. 4), where the most lateral contacts had the highest current. For precurved designs, electrodes in the basal turn were closest to the LW and had the highest current (20,34,58). Whereas straight arrays showed wide variability in scalar location at the round window (59), then transition from PM (lowest current) to LW (high current) two to three channels beyond the round window (19).

Interestingly, we found that the SlimJ device had significantly more deactivated electrodes than the Mid-Scala device. This is consistent with reports that straight arrays have more extracochlear electrodes (34,60–62) and are more prone to electrode migration (34,60,61) than precurved designs. However, our numbers for both devices were considerably higher than the 1 to 24% commonly reported in the literature (61,63–65).

Extracochlear electrodes occur in 1 to 13% of patients and cause inadequate auditory stimulation when activated (34,60–62). They may result from partial array insertion during surgery or movement of the array after implantation. Just 10% of deactivated electrodes were confirmed as extracochlear in our review. However, this is likely an underestimate due to using plain x-ray rather than computed tomography scans to check electrode position. In addition, electrode migration after initial imaging cannot be ruled out as repeat x-rays are typically only requested after trauma or large unexplained changes in performance. To accurately detect migration and extracochlear electrodes, routine monitoring of x-rays, computed tomographies or more practically, electrical field imaging (EFI) (66,67) is required to compare with intraoperative results. As electrode movement has implications for sound quality, the ability to monitor electrode position may help detect changes in placement that may require programming modifications.

The most common reason in our series for electrode deactivation was poor auditory perception (Supplementary Table 2, http://links.lww.com/MAO/B775). Poor perception may be related to physiological factors, such as anomalies in cochlear morphology or duration of deafness and survival of the spiral ganglion cells (2,68), or electrode position. Active electrodes near the round window, or outside the cochlea, may lead to higher current levels, sound distortion, extracochlear shunting of current, atypical loudness growth, and nonauditory perception (59). In addition, sound quality may change with biological changes, like fibrous tissue or bone growth over portions of the electrode (69). Importantly, despite audiologists using the same basic programming principles, individual tolerances will vary for when to deactivate electrodes. Although the decision to modify programming typically reflects subjective feedback or tests, it is important not to overlook the use of objective measurements like imaging, EFI, or neural responses, particularly in instances where performance was unexpectedly poor.

### Implant Integrity

This series reviews the performance of Advanced Bionics Ultra V1 implants for which a known fault exists (40). This fault affects both devices and is caused by reduced electrical output. To accommodate for the associated loss of sound, programming may include increasing threshold and comfort levels, or deactivating affected electrodes. To our knowledge, all patients with confirmed faults were removed. Unfortunately, impedances alone may not reliably detect all early failures (41). Without routine EFI, we cannot rule out the possibility that hearing outcomes or programming may be affected for a minority of the remaining patients in this series.

### Limitations

The tests used for hearing outcomes may not be sufficiently sensitive to detect differences in performance and listening effort. BKB sentences are the clinical standard in the United Kingdom; however, they offer greater context for top-down processing and are prone to ceiling effects. We are also unable to elaborate on how scalar location affects programming without access to higher-resolution imaging.

## CONCLUSIONS

HP was possible with both modern Advanced Bionics array designs but continues to be better with the straight electrode. Overall hearing outcomes were similar between the SlimJ and Mid-Scala arrays. Regardless of array, patients with preoperative hearing within EAS criteria performed better than more traditional CI candidates. “Traditional” CI candidates did not benefit more from a precurved compared to a straight electrode. However, there was less variation in performance for difficult listening conditions with the Mid-Scala device. Further information is required to understand the variability in basal electrode stimulation to determine if programming adjustments are required to optimize performance.

## Supplementary Material

SUPPLEMENTARY MATERIAL
