# Supplementary material for: A Multicenter Comparison of 1-yr Functional Outcomes and Programming Differences Between the Advanced Bionics Mid-Scala and SlimJ Electrode Arrays
Source: Otol Neurotol. 2023 Oct 27;44(10):e730–8. doi: 10.1097/MAO.0000000000004048 (PMC10662583; doi:10.1097/MAO.0000000000004048)
Supplement: SUPPLEMENTARY MATERIAL [file on-44-e730-s001.docx]

**Abstract**

**Objective:** To determine if there is a difference in hearing outcomes or stimulation levels between Advanced Bionics straight and pre-curved arrays.

**Study design:** Retrospective chart review across three implant centres.

**Setting:** Tertiary centres for cochlear and auditory brainstem implantation.

**Patients:** 115 paediatric and 205 adult cochlear implants (CIs) were reviewed. All patients were implanted under the National Institute for Health and Care Excellence 2009 guidelines with a HiRes Ultra SlimJ or Mid-Scala electrode array.

**Main outcome measures:** Hearing preservation following implantation, as well as CI-only listening scores for Bamford-Kowal-Bench (BKB) sentences were compared one-year post-implantation. Stimulation levels for threshold and comfort levels were also compared one-year post-implantation.

**Results:** Hearing preservation was significantly better with the SlimJ compared to the Mid-Scala electrode array. BKB outcomes were not significantly different between the two arrays in any listening condition. Stimulation levels were not different between arrays, but did vary across electrode contacts. At least one electrode was deactivated in 33% of implants, but was more common for the SlimJ device.

**Conclusion:** Modern straight and pre-curved arrays from Advanced Bionics did not differ in hearing performance or current requirements. Although hearing preservation was possible with both devices, the SlimJ array would still be the preferred electrode in cases where hearing preservation was a priority. Unfortunately, the SlimJ device was also prone to poor sound perception on basal electrodes. Further investigation is needed to determine if deactivated electrodes are associated with electrode position/migration, and if programming changes are needed to optimise the use of these high frequency channels.

**Introduction**

A cochlear implant (CI) is an implantable hearing device that provides sound through electrical stimulation. The design of a CI electrode array influences where the device is located within the cochlea, and can impact the amount of stimulation (current) required to reach audibility^1–3^, the risk of cochlear damage^4^, and hearing performance after implantation^5,6^.

Pre-curved arrays are designed to curl toward the modiolus in an effort to keep electrical stimulation close to the remaining auditory-neural tissues^7–9^. This perimodiolar (PM) placement helps minimise the amount of current needed to activate the auditory nerve,^1,2,8,10^ and potentially offers more focused stimulation^11^. Given the relationship between current, impedances and compliance, minimising current may be advantageous for maintaining stimulation rate, sound quality and battery life^10,12^. Additionally, electrodes positioned closer to the modiolus have been correlated with better word recognition^5,6^, improved electrode discrimination^13,14^ and reduced listening effort^13^. However, cochlear tissue damage may be more prevalent with PM designs due to their higher risk of tip fold-over and scalar translocation^4,15–18^.

In contrast, lateral wall (LW) arrays are ‘straight’ electrodes designed to sit away from the modiolus. They are further from the neural tissue and may require more current for auditory stimulation^1,19,20^. However, the simpler array design and insertion process helps reduce the risk of cochlear damage, and the incidence of translocation^21,22^. Minimising cochlear damage has implications for hearing preservation (HP), which has historically been better for LW designs^23,24^, and may be beneficial in challenging listening environments when using electro-acoustic stimulation (EAS)^25–27^.

Conventional evidence favours PM arrays for ‘traditional’ more severely deafened CI candidates, and LW arrays for those with more hearing^28^. However, with the advent of soft surgery techniques and less traumatic electrode designs, HP rates have improved for PM arrays^22,29^. Implant centres must now weigh the risks and benefits of modern array designs to determine if the benefits associated with modiolar proximity are still relevant^5,6,13,14^.

With this retrospective analysis of our implanted population, we compared the Advanced Bionics SlimJ and Mid-Scala electrodes to determine if conventional evidence holds true for these devices. Both arrays have demonstrated structural preservation in temporal bones which is important for HP^30^. However, there are still design differences that may be important to electrode selection. The SlimJ is a LW electrode with a narrower diameter than the Mid-Scala device^31^. Whereas the Mid-Scala is a pre-curved electrode, that does not hug the modiolus closely. Instead, it floats in the ‘middle’ of the scala tympani^22^ to offer a flexible free-fit with minimal damage^32–34^. To date, studies have compared histologic and radiological outcomes between the Mid-Scala and SlimJ electrodes^30^, but few studies have compared patient outcomes^18^.

For modern straight and pre-curved Advanced Bionics arrays, we aimed to investigate 1) if there was a difference in HP, 2) if hearing outcomes supported conventional choices for PM vs LW arrays, and 3) if the Mid-Scala device required lower current levels than the SlimJ device due to its position closer to the modiolus. We hypothesised that HP would be superior with the LW electrode, and that electrical hearing would be better with the pre-curved electrode.

**Methods**

A multi-centre retrospective chart review across three UK CI centres was conducted for 63 paediatric patients (115 implants) and 204 adult patients (205 implants) implanted between January 2016 and November 2018 (Table 1). This was registered as an audit and no formal ethics approval was required at our centres (PRN:7724).

All patients were implanted with Advanced Bionics HiRes Ultra Mid-Scala or SlimJ electrodes under the National Institute for Health and Care Excellence (NICE) 2009 guidelines^35^. Hearing thresholds before and after CI surgery, aetiology and age of onset for hearing loss were collected for each patient.

Low frequency pre-operative hearing thresholds at 250 and 500 Hz were used to differentiate between more severely deafened patients and potential EAS candidates. Thresholds for 125 Hz were not routinely measured, so could not be included in comparisons of hearing thresholds or HP.

The distribution of pre-operative hearing at 250 and 500 Hz was normal for SlimJ and Mid-Scala recipients (p>0.05, Kolmorogov Smirnoff) (Supplemental Figure 1, http://links.lww.com/MAO/B771). There was no significant difference between devices for pre-operative hearing at 250 (average: 79.0 dB HL SlimJ, 82.5 dB HL Mid-Scala; t=-1.06, p=0.29) or 500 Hz (average: 86.9 dB HL SlimJ, 94.6 dB HL Mid-Scala; t=-0.79, p=0.43), indicating that pre-operative hearing did not bias electrode selection.

*Hearing preservation*

Degree of HP (% HP) and HP classification at low frequencies was calculated using a modified HEARRING group formula^23,36,37^, by including only 250-500 Hz thresholds. To ensure no audiograms were missed, hearing tests completed up to 6 months post-implantation were collected, however most were completed within 3 months of surgery (n=127 out of 131).

*Hearing outcomes*

Bamford-Kowal-Bench (BKB) sentence (% words correct) scores were collected for CI-only listening conditions one-year post-implantation (12 +/- 4 months) for adult recipients. Scores were collected for BKB sentences when listening to a male speaker in quiet, female speaker in quiet, male speaker in noise and female speaker in noise conditions. All speech in noise testing used pink noise at +10 dB signal-to-noise ratio (SNR).

To check if more severely deafened CI candidates should receive the pre-curved array and those with better hearing the LW array, BKB scores were compared based on pre-operative hearing for 250 and 500 Hz separately. Patients were grouped into the following ranges for each frequency: a) hearing likely to benefit from EAS (≤70 dB HL) and b) hearing unlikely to benefit from EAS (>70 dB HL). Due to the limited use of combined stimulation, comparisons for active EAS components could not be made.

*Programming details*

Threshold level, comfort level, pulse width and number of deactivated electrodes were collected one-year post-implantation. Threshold and comfort levels were presented in charge units (cu), as listed in the SoundWave software. This allowed for direct comparison of clinical MAPs, as cu already incorporates pulse width [cu=(microAmps x pulse width)/79]. Levels were also converted to charge [nC/phase=cu*79] to enable comparison with other manufacturers. Soundfield aided thresholds (SFAT) one-year post-implantation confirmed that sound detection was within the clinically recommended range (20-30 dB HL^38,39^) for most patients (Supplementary Figure 2, http://links.lww.com/MAO/B772), indicating acceptable threshold levels in most cases. Thresholds were measured at centre 1 and 2, but left at default (10% of comfort levels) for most adults at centre 3. Threshold levels were also set to default for most paediatric patients at centre 1 and 2.

*Exclusions (Table 1)*

Patients who did not use spoken language as their primary mode of communication (5 SlimJ, 7 Mid-Scala) were excluded from all comparison. Patients were also removed if they did not attend their one-year appointment (6 SlimJ, 9 Mid-Scala), or showed signs of V1 failure^40,41^ (6 SlimJ, 23 Mid-Scala).

*Statistics*

All tests were completed using SPSS 26 statistics package^42^. Normality was checked using the Kolmorogov-Smirnoff test. Arrays were compared to check for differences in pre-operative hearing (independent T-test), HP (Mann-Whitney U and Kruskal Wallis tests), overall BKB outcomes (Mann-Whitney U test), and variance in BKB outcomes (Levene’s Test). Additionally, interactions were checked for BKB outcome using univariate ANOVA, with factors electrode (SlimJ vs Mid-Scala) and hearing threshold (≤70 dB HL vs >70 dB HL) for 250 and 500 Hz separately. Distribution of threshold and comfort levels were tested separately using repeated measures ANOVA, with factors channel (electrodes 1-15, electrode 16 excluded) and implant centre (1 or 2, 3 excluded). Threshold and comfort levels were significant when tested against Mauchley’s test of sphericity, therefore the Greenhouse Geisser correction was used. Finally, the number of deactivated channels was analysed with the chi-square test.

**Results**

***Do arrays differ in HP?***

Post-operative hearing was measured for 131 ears (73 SlimJ, 58 Mid-Scala). Degree of HP was not normally distributed (Kolmogorov-Smirnoff = 0.13, p<0.001). Overall, degree of HP was better for the SlimJ device (average HP: 54% SlimJ, 41% Mid-Scala, Mann-Whitney U=1675, p=0.04)(Figure 1), with no effect of centre (Kruskal Wallis = 1.04, p=0.59).

The degree of HP was better for EAS candidates (average HP 57%) compared to non-EAS candidates (average HP 43%), with a significant effect of electrode (Mann-Whitney U=1548, p=0.028). The electrode effect showed better HP with the SlimJ device for EAS candidates (average HP: 66% SlimJ, 42% Mid-Scala; Mann-Whitney U=151.5, p=0.006), but no significant difference for non-EAS candidates (average HP: 46% SlimJ, 40% Mid-Scala; Mann-Whitney U=760, p=0.462). Despite at least some HP in most cases, EAS was active for only 7 adult and 8 paediatric implants one-year post-implantation.

***Do hearing outcomes differ between arrays?***

BKB outcomes were skewed for both arrays in all test conditions (Kolmorogov-Smirnoff, p≤0.007), and were limited by ceiling effects (Figure 2).

Overall, speech perception was not different between the arrays in any BKB test condition [average: male speaker in quiet (78% SlimJ, 73% Mid-Scala; Mann-Whitney U=1960.0, p=0.26), female speaker in quiet (79% SlimJ, 77% Mid-Scala; Mann-Whitney U=892.5, p=0.80), male speaker in noise (61% SlimJ, 71% Mid-Scala; Mann-Whitney U=545.5, p=0.23), female speaker in noise (65% SlimJ, 59% Mid-Scala; Mann-Whitney U=118.0, p=0.56)]. However, there was a greater spread in performance with a male speaker in noise for the SlimJ device (F=7.965, p=0.006; Levene’s test; Figure 2a). The spread of performance was not significantly different for tests in quiet, and could not be compared for the female speaker in noise due to small sample sizes.

To check if one electrode was better for speech in noise, we examined how performance reduces when listening in noise compared to when listening in quiet. The average decrease in performance was similar between the two arrays for both speakers [average percentage point decrease: male (27 SlimJ, 20 Mid-Scala; Mann-Whitney U=534, p=0.297), female (24 SlimJ, 27 Mid-Scala; Mann-Whitney U=123.5, p=0.682)]. However, there was greater variability in outcomes for the male speaker with the SlimJ electrode (Levene’s test, F=8.396, p=0.005; Figure 2b).

*Hearing performance vs. pre-operative thresholds*

To test if LW arrays should be the preferred choice for EAS candidates (i.e., pre-operative low frequency hearing better than 70 dB HL), pre-operative hearing was compared separately for 250 and 500 Hz. For each frequency, BKB results were compared between EAS candidates (≤70 dB HL), and non-EAS candidates (>70 dB HL) (Figure 3).

Regardless of electrode, patients with pre-operative hearing ≤70 dB HL had better BKB scores when listening to a male speaker in quiet and in noise. This held true for 250 and 500 Hz [average ≤70 dB HL vs >70 dB HL for: 250 Hz in quiet (84% vs 71%; ANOVA, F(1,129)=8.15, p=0.005), 250 Hz in noise (74% vs 60%; ANOVA, F(1,57)=4.48, p=0.04), 500 Hz in quiet (88% vs 73%; ANOVA, F(1,129)=6.64, p=0.01), 500 Hz in noise (81% vs 62%; ANOVA, F(1,57)=6.72, p=0.01)].

There was also an interaction when listening to a male speaker in noise between electrode and frequency (ANOVA, F(1,57)=4.06, p=0.049), where performance was worse for SlimJ recipients, but only when pre-operative hearing was >70 dB HL at 500 Hz (average: 54% SlimJ, 71% Mid-Scala). A similar trend was observed for 250 Hz, but was not significant (average: 53% SlimJ, 67% Mid-Scala; ANOVA, F(1,57)=3.32, p=0.07). However, this interaction is likely attributed to 6 SlimJ recipients who experienced significant difficulty when listening in noise (Figure 2b).

When listening to a female speaker in quiet, BKB scores were similar regardless of pre-operative hearing and electrode (ANOVA, p>0.05). Unfortunately, due to limited sample size the female speaker in noise condition could not be analysed. Regardless of electrode, patients with better pre-operative hearing at 250 or 500 Hz tended to have higher BKB scores only when listening to the male speaker.

***Does the Mid-Scala device require lower stimulation levels?***

One-year programming MAPs were collected for 131 SlimJ and 133 Mid-Scala implants. Deactivated electrodes were more common for the SlimJ device, with 60 (46%) SlimJ and 42 (32%) Mid-Scala devices having at least one electrode turned off (Chi square=4.69, N=265, p=0.03). This trend was common across all centres, as well as for adult and paediatric recipients (Supplementary Table 1, http://links.lww.com/MAO/B774). Basal electrodes were most frequently deactivated for both arrays. The most common reason was inadequate sound perception, including poor sound detection or loudness growth (reasons in Supplementary Table 2, http://links.lww.com/MAO/B775).

*Current levels*

Average threshold and comfort levels are shown separately for each centre due to differences in programming (Figure 4, Supplementary Figure 3, http://links.lww.com/MAO/B773). For adults (Figure 4a), there was no significant main effect of electrode or centre for threshold or comfort level. There was, however, a significant effect for electrode channel (electrodes 1-15) on threshold (F(2.8, 253.5)=7.04, p<0.001), and comfort level (F(3.3, 295.0)=8.8, p<0.001). Similarly, there was an interaction for comfort level between channel and array (F(3.3, 295.0)=4.9, p=0.001), where arrays had different comfort levels, but only for basal channels. However, to address our question, overall current requirements were similar for both devices.

Comfort levels for centre 3 appeared elevated compared to centres 1 and 2 (Figure 4a). However, centre 3 was not included in the ANOVA due to using default (10% of comfort levels) rather than measured thresholds.

For paediatric patients, there was no significant main effect of centre or electrode for threshold or comfort level. There was, however, a significant effect for electrode channel on threshold (F(3.6, 284)=11.6, p<0.001) and comfort level (F(3.7, 290)=29.9, p<0.001) (Figure 4b). No interaction between array and channel was observed, although this may be related to reliance on objective measures rather than behavioural based programming approaches.

**Discussion**

***Do arrays differ in HP?***

HP was better with the SlimJ compared to the Mid-Scala array. This aligns with previous studies showing better HP for LW than PM electrodes^23,43,44^. Although HP may be better with LW arrays, it is still often possible to preserve hearing with pre-curves designs^45,46^. Recent studies have even shown similar rates of HP between PM and LW arrays^22,47^. However, HP is multi-factorial, with factors beyond electrode design under the surgeons’ control.

Pre-operative hearing also impacted HP, where those with better hearing had better HP. Here, the best HP results were for SlimJ recipients whose pre-operative hearing fell within EAS candidacy. This relationship between pre-operative hearing and HP has previously been reported^44,48^. However, not all studies have supported this correlation^49^.

Despite the clinical importance, there is limited consistency when reporting HP. HP must be interpreted cautiously, particularly when comparing across different measurement approaches. ‘Functional hearing’ preservation will vary depending on the tolerances used for threshold change or percentage change measurements, and by which frequencies were included in the calculations^37^.

***Do hearing outcomes differ between arrays?***

We found no significant difference in average BKB performance between arrays. There was no clear benefit for Mid-Scala recipients with more ‘traditional’ pre-operative hearing thresholds, nor was there a benefit for EAS candidates implanted with the SlimJ device. These data suggest that modern Advanced Bionics arrays do not differ in aided hearing outcomes based on pre-operative hearing.

This aligns with recent studies reporting no significant difference on sentence or disyllabic tests between modern pre-curved and straight arrays^13,50–55^. However, some studies have reported better performance for PM arrays on electrode discrimination^13^, listening effort^13^, and consonant nucleus consonant tests^47,51,55,56^. In the largest review to date, PM arrays outperformed straight arrays on AzBio sentences only after considering additional factors^47^. Although we did find an effect for Mid-Scala recipients with poorer pre-operative hearing, this was limited only to the distribution of BKB outcomes when listening to a male speaker in noise. This could point to a potential benefit of the Mid-Scala array in challenging listening environments for more traditional candidates. However, it may also be skewed by 6 SlimJ recipients who had disproportionately more difficulty (drop of >40% points) in noise compared to quiet listening conditions. This degree of difficulty was not observed for any Mid-Scala recipients, nor could it be explained by pre-lingual/post-lingual deafness, aetiology, age at implant, SFAT, number of deactivated electrodes, or threshold/comfort levels.

When comparing outcomes between potential EAS candidates, the similarity in performance between arrays may be related to the limited use of combined stimulation. Historically, benefit from HP has been associated with EAS use^21,26,27,57^. Unfortunately, despite successful HP, only 15 of our implants had active EAS components one-year post-implantation. As most of these were paediatric recipients with no BKB testing, we were unable to compare hearing outcomes for this group.

Given similarities in performance between modern straight and pre-curved devices, electrode selection may be driven by factors such as cochlear anatomy or aetiology rather than hearing thresholds. We are, however, mindful that BKB sentences may not be sensitive enough to detect potential benefits of HP or modiolar proximity. Single word or non-speech tests may better highlight performance differences between array designs. Additionally, investigation into the limited use of EAS is needed to determine what barriers led to such poor uptake of combined stimulation.

***Does the Mid-Scala device require lower stimulation levels?***

There was clear variation in current requirements across electrode channels, but no difference between arrays for overall current levels. This could be a reflection of the Mid-Scala array placement, where cochlear position ranges from entirely mid-scalar to mostly lateral wall depending on cochlear size^20,34,58^. However, these results are consistent with recent studies showing similar charge requirements between modern PM and LW arrays^51,56^.

Current levels by channel reflect previously published data about scalar location (Figure 4), where the most lateral contacts had the highest current. For pre-curved designs, electrodes in the basal turn were closest to the lateral wall and had the highest current^20,34,58^. Whereas straight arrays showed wide variability in scalar location at the round window^59^, then transition from perimodiolar (lowest current) to lateral wall (high current) 2-3 channels beyond the round window^19^.

Interestingly, we found that the SlimJ device had significantly more deactivated electrodes than the Mid-Scala device. This is consistent with reports that straight arrays have more extra-cochlear electrodes^34,60–62^ and are more prone to electrode migration^34,60,61^ than pre-curved designs. However, our numbers for both devices were considerably higher than the 1-24% commonly reported in the literature^61,63–65^.

Extra-cochlear electrodes occur in 1-13% of patients, and cause inadequate auditory stimulation when activated^34,60–62^. They may result from partial array insertion during surgery, or movement of the array after implantation. Just 10% of deactivated electrodes were confirmed as extra-cochlear in our review. However, this is likely an underestimate due to using plain X-ray rather than CT scans to check electrode position. Additionally, electrode migration after initial imaging cannot be ruled out as repeat X-rays are typically only requested following trauma or large unexplained changes in performance. To accurately detect migration and extra-cochlear electrodes, routine monitoring of X-rays, CTs, or more practically, electrical field imaging (EFI)^66,67^, is required to compare with intra-operative results. As electrode movement has implications for sound quality, the ability to monitor electrode position may help detect changes in placement that may require programming modifications.

The most common reason in our series for electrode deactivation was poor auditory perception (Supplementary Table 2, http://links.lww.com/MAO/B775). Poor perception may be related to physiological factors; such as anomalies in cochlear morphology or duration of deafness and survival of the spiral ganglion cells^2,68^; or electrode position. Active electrodes near the round window, or outside the cochlea, may lead to higher current levels, sound distortion, extra-cochlear shunting of current, atypical loudness growth and non-auditory perception^59^. Additionally, sound quality may change with biological changes, like fibrous tissue or bone growth over portions of the electrode^69^. Importantly, despite audiologists following the same basic programming principles, individual tolerances will vary for when to deactivate electrodes. While the decision to modify programming typically reflects subjective feedback or tests, it is important not to overlook the use of objective measurements like imaging, EFI or neural responses, particularly in instances where performance was unexpectedly poor.

***Implant integrity***

This series reviews performance of Advanced Bionics Ultra V1 implants for which a known fault exists^40^. This fault affects both devices, and is caused by reduced electrical output. To accommodate for the associated loss of sound, programming may include increasing threshold and comfort levels, or deactivating affected electrodes. To our knowledge all patients with confirmed faults were removed. Unfortunately, impedances alone may not reliably detect all early failures^41^. Without routine EFI, we cannot rule out the possibility that hearing outcomes or programming may be affected for a minority of the remaining patients in this series.

***Limitations***

The tests used for hearing outcomes may not be sufficiently sensitive to detect differences in performance and listening effort. BKB sentences are the clinical standard in the UK, however, they offer greater context for top down processing and are prone to ceiling effects. We are also unable to elaborate on how scalar location affects programming without access to higher resolution imaging.

***Conclusion***

Hearing preservation was possible with both modern Advanced Bionics array designs, but continues to be better with the straight electrode. Overall hearing outcomes were similar between the SlimJ and Mid-Scala arrays. Regardless of array, patients with pre-operative hearing within EAS criteria performed better than more traditional CI candidates. ‘Traditional’ CI candidates did not benefit more from a pre-curved compared to a straight electrode. However, there was less variation in performance for difficult listening conditions with the Mid-Scala device. Further information is required to understand the variability in basal electrode stimulation to determine if programming adjustments are required to optimise performance.

**References:**

1. Davis TJ, Zhang D, Gifford RH, Dawant BM, Labadie RF, Noble JH. Relationship between electrode-To-modiolus distance and current levels for adults with cochlear implants. *Otol Neurotol*. 2016;37(1):31-37. doi:10.1097/MAO.0000000000000896

2. Saunders E, Cohen L, Aschendorff A, et al. Threshold, comfortable level and impedance changes as a function of electrode-modiolar distance. *Ear Hear*. 2002;23(1 SUPPL.). doi:10.1097/00003446-200202001-00004

3. Cohen LT, Saunders E, Clark GM. Psychophysics of a prototype peri-modiolar cochlear implant electrode array. *Hear Res*. 2001;155(1-2):63-81. doi:10.1016/S0378-5955(01)00248-9

4. Dhanasingh A, Jolly C. An overview of cochlear implant electrode array designs. *Hear Res*. 2017;356:93-103. doi:10.1016/j.heares.2017.10.005

5. Holden LK, Finley CC, Firszt JB, et al. Factors Affecting Open-Set Word Recognition in Adults With Cochlear Implants. *Ear Hear*. 2013;34(3):342-360. doi:10.1097/AUD.0b013e3182741aa7

6. Gordin A, Papsin B, James A, Gordon K. Evolution of cochlear implant arrays result in changes in behavioral and physiological responses in children. *Otol Neurotol*. 2009;30(7):908-915. doi:10.1097/MAO.0b013e3181b236b0

7. Shepherd RK, Hatsushika S, Clark GM. Electrical stimulation of the auditory nerve: The effect of electrode position on neural excitation. *Hear Res*. 1993;66(1):108-120. doi:10.1016/0378-5955(93)90265-3

8. Cohen LT. Practical model description of peripheral neural excitation in cochlear implant recipients: 2. Spread of the effective stimulation field (ESF), from ECAP and FEA. *Hear Res*. 2009;247(2):100-111. doi:10.1016/j.heares.2008.11.004

9. Van Wermeskerken GKA, Van Olphen AF, Graamans K. Imaging of electrode position in relation to electrode functioning after cochlear implantation. *Eur Arch Oto-Rhino-Laryngology*. 2009;266(10):1527-1531. doi:10.1007/s00405-009-0939-2

10. Jeong J, Kim M, Heo JH, et al. Intraindividual comparison of psychophysical parameters between perimodiolar and lateral-type electrode arrays in patients with bilateral cochlear implants. *Otol Neurotol*. 2015;36(2):228-234. doi:10.1097/MAO.0000000000000672

11. Cohen LT, Saunders E, Knight MR, Cowan RSC. Psychophysical measures in patients fitted with Contour^TM^ and straight Nucleus electrode arrays. *Hear Res*. 2006;212(1-2):160-175. doi:10.1016/j.heares.2005.11.005

12. Saoji AA, Adkins WJ, Olund AP, Nelson-Bakkum ER, Koka K. Effect of exceeding compliance voltage on speech perception in cochlear implants. *Hear Res*. 2021;400:108112. doi:10.1016/j.heares.2020.108112

13. Ramos-De-Miguel A, Carlos Falcón-González J, Ramos-Macias A. Analysis of Neural Interface When Using Modiolar Electrode Stimulation. Radiological Evaluation, Trans-Impedance Matrix Analysis and Effect on Listening Effort in Cochlear Implantation. Published online 2021. doi:10.3390/jcm10173962

14. Macias AR, Zaballos MTP, De Miguel AR, Paz JC. Importance of perimodiolar electrode position for psychoacoustic discrimination in cochlear implantation. *Otol Neurotol*. 2017;38(10):e429-e437. doi:10.1097/MAO.0000000000001594

15. Durakovic N, Kallogjeri D, Wick CC, McJunkin JL, Buchman CA, Herzog J. Immediate and 1-Year Outcomes with a Slim Modiolar Cochlear Implant Electrode Array. *Otolaryngol - Head Neck Surg (United States)*. 2020;162(5):731-736. doi:10.1177/0194599820907336

16. Jwair S, Prins A, Wegner I, Stokroos RJ, Versnel H, Thomeer HGXM. Scalar Translocation Comparison Between Lateral Wall and Perimodiolar Cochlear Implant Arrays - A Meta-Analysis. *Laryngoscope*. 2021;131(6):1358-1368. doi:10.1002/lary.29224

17. Finley CC, Holden TA, Holden LK, et al. Role of electrode placement as a contributor to variability in cochlear implant outcomes. *Otol Neurotol*. 2008;29(7):920-928. doi:10.1097/MAO.0b013e318184f492

18. Harris MS, Koka K, Riggs WJ, et al. Can Electrocochleography Help Preserve Hearing after Cochlear Implantation with Full Electrode Insertion? *Otol Neurotol*. 2022;43(7):789-796. doi:10.1097/MAO.0000000000003588

19. Degen CV, Büchner A, Kludt E, Lenarz T. Effect of Electrode to Modiolus Distance on Electrophysiological and Psychophysical Parameters in CI Patients With Perimodiolar and Lateral Electrode Arrays. *Otol Neurotol*. 2020;41(9):e1091-e1097. doi:10.1097/MAO.0000000000002751

20. Long CJ, Holden TA, McClelland GH, et al. Examining the electro-neural interface of cochlear implant users using psychophysics, CT scans, and speech understanding. *JARO - J Assoc Res Otolaryngol*. 2014;15(2):293-304. doi:10.1007/s10162-013-0437-5

21. Wanna GB, Noble JH, Carlson ML, et al. Impact of electrode design and surgical approach on scalar location and cochlear implant outcomes. *Laryngoscope*. 2014;124(S6):S1-S7. doi:10.1002/lary.24728

22. Woodson E, Smeal M, Nelson RC, Haberkamp T, Sydlowski S. Slim Perimodiolar Arrays Are as Effective as Slim Lateral Wall Arrays for Functional Hearing Preservation after Cochlear Implantation. *Otol Neurotol*. 2020;41(6):e674-e679. doi:10.1097/MAO.0000000000002622

23. Skarzynski H, Lorens A, Matusiak M, Porowski M, Skarzynski PH, James CJ. Partial deafness treatment with the nucleus straight research array cochlear implant. *Audiol Neurotol*. 2012;17(2):82-91. doi:10.1159/000329366

24. Mistrík P, Jolly C, Sieber D, Hochmair I. Challenging aspects of contemporary cochlear implant electrode array design Production and Hosting by Elsevier on behalf of KeAi. *World J Otorhinolaryngol Neck Surg*. 2017;3:192-199. doi:10.1016/j.wjorl.2017.12.007

25. Gifford RH, Dorman MF, Brown CA. Psychophysical properties of low-frequency hearing: Implications for perceiving speech and music via electric and acoustic stimulation. In: *Cochlear Implants and Hearing Preservation*. Vol 67. S. Karger AG; 2009:51-60. doi:10.1159/000262596

26. Turner C, Gantz BJ, Reiss L. Integration of acoustic and electrical hearing. *J Rehabil Res Dev*. 2008;45(5):769-778. doi:10.1682/JRRD.2007.05.0065

27. Gfeller KE, Olszewski C, Turner C, Gantz B, Oleson J. Music perception with cochlear implants and residual hearing. *Audiol Neurotol*. 2006;11(SUPPL. 1):12-15. doi:10.1159/000095608

28. Gibson P, Boyd P. Optimal electrode design: Straight versus perimodiolar. *Eur Ann Otorhinolaryngol Head Neck Dis*. 2016;133:S63-S65. doi:10.1016/j.anorl.2016.04.014

29. Fraysse B, Macías ÁR, Sterkers O, et al. Residual hearing conservation and electroacoustic stimulation with the nucleus 24 contour advance cochlear implant. *Otol Neurotol*. 2006;27(5):624-633. doi:10.1097/01.mao.0000226289.04048.0f

30. Downing M. Electrode designs for protection of the delicate cochlear structures. *J Int Adv Otol*. 2018;14(3):401-403. doi:10.5152/iao.2018.6461

31. Rivas A, Yawn RJ, Kim AH, et al. A New Lateral Wall Electrode: Evaluation of Surgical Handling, Radiographic Placement, and Histological Appraisal of Insertion Trauma. *Otol Neurotol*. 2019;40(5S Suppl 1):S23-S28. doi:10.1097/MAO.0000000000002210

32. Briggs R, Tykocinski M, Saunders E, et al. Surgical implications of perimodiolar cochlear implant electrode design: avoiding intracochlear damage and scala vestibuli insertion. *Cochlear Implants Int*. 2001;2(2):135-149. doi:10.1179/cim.2001.2.2.135

33. Hassepass F, Bulla S, Maier W, et al. The new mid-scala electrode array: A radiologic and histologic study in human temporal bones. *Otol Neurotol*. 2014;35(8):1415-1420. doi:10.1097/MAO.0000000000000412

34. Dietz A, Gazibegovic D, Tervaniemi J, Vartiainen VM, Löppönen H. Insertion characteristics and placement of the Mid-Scala electrode array in human temporal bones using detailed cone beam computed tomography. *Eur Arch Oto-Rhino-Laryngology*. 2016;273(12):4135-4143. doi:10.1007/s00405-016-4099-x

35. *Cochlear Implants for Children and Adults with Severe to Profound Deafness | Guidance | NICE*. NICE; 2009.

36. O’Leary S, Briggs R, Gerard JM, et al. Intraoperative Observational Real-time Electrocochleography as a Predictor of Hearing Loss After Cochlear Implantation: 3 and 12 Month Outcomes. *Otol Neurotol*. 2020;41(9):1222-1229. doi:10.1097/MAO.0000000000002773

37. Adunka OF, Gantz BJ, Dunn C, et al. Minimum Reporting Standards for Adult Cochlear Implantation. 2018;159(2):215-219. doi:10.1177/0194599818764329.Minimum

38. Holder JT, Holcomb MA, Snapp H, et al. Guidelines for Best Practice in the Audiological Management of Adults Using Bimodal Hearing Configurations. *Otol Neurotol Open*. 2022;2(2):e011. doi:10.1097/ONO.0000000000000011

39. de Graaff F, Lissenberg-Witte BI, Kaandorp MW, et al. Relationship Between Speech Recognition in Quiet and Noise and Fitting Parameters, Impedances and ECAP Thresholds in Adult Cochlear Implant Users. *Ear Hear*. 2020;41(4):935-947. doi:10.1097/AUD.0000000000000814

40. Voluntary Field Corrective Action of HiRes Ultra and Ultra 3D | Advanced Bionics. Accessed June 9, 2021. https://advancedbionics.com/com/en/home/about-us/news/press-releases/2020/voluntary-field-corrective-action-hires-ultra-and-ultra-3d.html

41. Eitutis ST, Tam YC, Roberts I, et al. Detecting and managing partial shorts in Cochlear implants: A validation of scalp surface potential testing. *Clin Otolaryngol*. 2022;47(6):641-649. doi:10.1111/coa.13963

42. IBM SPSS Statistics for Windows, Version 26.0. Published online 2019.

43. Perkins EL, Labadie RF, O’Malley M, et al. The Relation of Cochlear Implant Electrode Array Type and Position on Continued Hearing Preservation. *Otol Neurotol*. 2022;43(6):e634-e640. doi:10.1097/MAO.0000000000003547

44. Ludwig S, Riemann N, Hans S, et al. Evaluation of hearing preservation in adults with a slim perimodiolar electrode. *Eur Arch Oto-Rhino-Laryngology*. 2021;1:3. doi:10.1007/s00405-021-06755-z

45. Woodson E, Nelson RC, Smeal M, Haberkamp T, Sydlowski S. Initial hearing preservation outcomes of cochlear implantation with a slim perimodiolar electrode array. *Cochlear Implants Int*. 2021;22(3):148-156. doi:10.1080/14670100.2020.1858553

46. Nassiri AM, Yawn RJ, Holder JT, et al. Hearing preservation outcomes using a precurved electrode array inserted with an external sheath HHS Public Access. *Otol Neurotol*. 2020;41(1):33-38. doi:10.1097/MAO.0000000000002426

47. Sharma RK, Smetak MR, Patro A, et al. Speech Recognition Performance Differences Between Precurved and Straight Electrode Arrays From a Single Manufacturer. *Otol Neurotol*. 2022;43(10):1149-1154. doi:10.1097/mao.0000000000003703

48. Balkany TJ, Connell SS, Hodges A V., et al. Conservation of residual acoustic hearing after cochlear implantation. *Otol Neurotol*. 2006;27(8):1083-1088. doi:10.1097/01.mao.0000244355.34577.85

49. Khoza-Shangase K, Gautschi-Mills K. Exploration of factors influencing the preservation of residual hearing following cochlear implantation. *South African J Commun Disord*. 2019;66(1):1-7. doi:10.4102/sajcd.v66i1.607

50. MacPhail ME, Connell NT, Totten DJ, et al. Speech Recognition Outcomes in Adults With Slim Straight and Slim Modiolar Cochlear Implant Electrode Arrays. *Otolaryngol - Head Neck Surg (United States)*. 2022;166(5):943-950. doi:10.1177/01945998211036339

51. Holder JT, Yawn RJ, Nassiri AM, et al. Matched Cohort Comparison Indicates Superiority of Precurved Electrode Arrays HHS Public Access. *Otol Neurotol*. 2019;40(9):1160-1166. doi:10.1097/MAO.0000000000002366

52. Doshi J, Johnson P, Mawman D, et al. Straight vs. Modiolar hugging electrodes – Does one perform better than the other? *Cochlear Implants Int*. 2015;16(S1):S33-S35. doi:10.1179/1467010014Z.000000000231

53. Schwartz N, Brown KD, Park LR. Audiologic Outcomes of Cochlear Implantation in Cochlear Malformations: A Comparative Analysis of Lateral Wall and Perimodiolar Electrode Arrays. *Otol Neurotol*. 2020;41(10):e1201-e1206. doi:10.1097/MAO.0000000000002833

54. Battmer RD, Scholz S, Gazibegovic D, Ernst A, Seidl RO. Comparison of a Mid Scala and a Perimodiolar Electrode in Adults: Performance, Impedances, and Psychophysics. *Otol Neurotol*. 2020;41(4):467-475. doi:10.1097/MAO.0000000000002579

55. Sturm JJ, Patel V, Dibelius G, Kuhlmey M, Kim AH. Comparative Performance of Lateral Wall and Perimodiolar Cochlear Implant Arrays. *Otol Neurotol*. 2021;42(4):532-539. doi:10.1097/MAO.0000000000002997

56. Park LR, Teagle HFB, Brown KD, Gagnon EB, Woodard JS, Buchman CA. Audiological Outcomes and Map Characteristics in Children with Perimodiolar and Slim Straight Array Cochlear Implants in Opposite Ears. *Otol Neurotol*. 2017;38(9):e320-e326. doi:10.1097/MAO.0000000000001539

57. Gifford RH, Dorman MF, Skarzynski H, et al. Cochlear implantation with hearing preservation yields significant benefit for speech recognition in complex listening environments. 2014;34(4):413-425. doi:10.1097/AUD.0b013e31827e8163.Cochlear

58. Frisch CD, Carlson ML, Lane JI, Driscoll CLW. Evaluation of a new mid-scala cochlear implant electrode using microcomputed tomography. *Laryngoscope*. 2015;125(12):2778-2783. doi:10.1002/lary.25347

59. Salcher R, Boruchov A, Timm M, et al. On the Intracochlear Location of Straight Electrode Arrays After Cochlear Implantation: How Lateral Are Lateral Wall Electrodes? *Otol Neurotol*. 2021;42(2):242-250. doi:10.1097/MAO.0000000000002880

60. Rader T, Baumann U, Stover T, et al. Management of cochlear implant electrode migration. *Otol Neurotol*. 2016;37(9):e341-e348. doi:10.1097/MAO.0000000000001065

61. Holder JT, Kessler DM, Noble JH, Gifford RH, Labadie RF. Prevalence of extracochlear electrodes: Computerized tomography scans, cochlear implant maps, and operative reports. *Otol Neurotol*. 2018;39(5):e325-e331. doi:10.1097/MAO.0000000000001818

62. Marlowe AL, Chinnici JE, Rivas A, Niparko JK, Francis HW. Revision Cochlear Implant Surgery in Children. *Otol Neurotol*. 2010;31(1):74-82. doi:10.1097/MAO.0b013e3181c29fad

63. Francis HW, Buchman CA, Visaya JM, et al. Surgical factors in pediatric cochlear implantation and their early effects on electrode activation and functional outcomes. *Otol Neurotol*. 2008;29(4):502-508. doi:10.1097/MAO.0b013e318170b60b

64. Zeitler DM, Lalwani AK, Roland JT, Habib MG, Gudis D, Waltzman SB. The effects of cochlear implant electrode deactivation on speech perception and in predicting device failure. *Otol Neurotol*. 2009;30(1):7-13. doi:10.1097/MAO.0b013e31818a08ba

65. Carlson ML, Neff BA, Sladen DP, Link MJ, Driscoll CL. Cochlear implantation in patients with intracochlear and intralabyrinthine schwannomas. In: *Otology and Neurotology*. Vol 37. Lippincott Williams and Wilkins; 2016:647-653. doi:10.1097/MAO.0000000000001016

66. De Rijk SR, Tam YC, Carlyon RP, Bance ML. Detection of Extracochlear Electrodes in Cochlear Implants with Electric Field Imaging/Transimpedance Measurements:: A Human Cadaver Study. *Ear Hear*. 2020;41(5):1196-1207. doi:10.1097/AUD.0000000000000837

67. de Rijk SR, Hammond-Kenny A, Tam YC, et al. Detection of Extracochlear Electrodes Using Stimulation-Current- Induced Non-Stimulating Electrode Voltage Recordings With Different Electrode Designs. *Otol Neurotol*. 2022;43(5):e548-e557. doi:10.1097/MAO.0000000000003512

68. Goldwyn JH, Bierer SM, Bierer JA. Modeling the electrode-neuron interface of cochlear implants: Effects of neural survival, electrode placement, and the partial tripolar configuration. *Hear Res*. 2010;268(1-2):93-104. doi:10.1016/j.heares.2010.05.005

69. Somdas MA, Li PMMC, Whiten DM, Eddington DK, Nadol JB. Quantitative evaluation of new bone and fibrous tissue in the cochlea following cochlear implantation in the human. *Audiol Neurotol*. 2007;12(5):277-284. doi:10.1159/000103208

**Figure Legends:**

**Supplementary Figure 1:** Pre-operative hearing thresholds at frequencies most commonly used for electro-acoustic stimulation (250 & 500 Hz) in adult cochlear implant recipients. Hearing thresholds are plotted as back-to-back histograms, where the size of the coloured bar reflects the number of individuals with hearing thresholds at each interval. SlimJ recipients (n=97) are in teal and Mid-Scala recipients (n=96) are in pink, with the mean (+) and median (☐) shown for each measurement. Pre-operative hearing for both frequencies and electrodes were within acceptable ranges for skewness and kertosis, and had normal distributions. There was no significant difference between electrode groups for pre-operative hearing at 250 Hz (t = -1.06, df = 190, p > 0.05) or at 500 Hz (t = -0,79, df = 189, p > 0.05).

**Supplementary Figure 2:** Soundfield aided thresholds (SFAT) at 9-12 month programming for a) adult patients and b) paediatric patients. The number of patients included at each frequency is shown for SlimJ (teal) and Mid-Scala (pink), with the mean (+) and median (☐) shown for each measurement. The data is plotted as back-to-back histograms, where size of the bar indicates the number of patients with the corresponding threshold in dB HL.

**Figure 1:** Percentage (%) hearing preservation following cochlear implant surgery. a) Hearing preservation shown by electrode, regardless of pre-operative hearing thresholds. b) Hearing preservation by electrode based on pre-operative hearing thresholds for 250 Hz. The number of patients is listed for SlimJ (teal) and Mid-Scala (pink) electrodes, along with the mean (+) and median (☐) for each measurement. (* p<0.05, Mann-Whitney U)

**Figure 2:** BKB results 12-months post activation for adult patients only. a) Results shown for each test condition. b) Difference in BKB scores when comparing noise and quiet conditions for the same speaker. % drop in noise refers to the percentage point difference between the noise and quiet condition. The data is plotted as back-to-back histograms, where the size of the coloured bars reflects the number of individuals with BKB scores in that % range. The number of measurements in each listening condition is listed for SlimJ (teal) and Mid-Scala (pink) electrodes, with the mean (+) and median (☐) shown for each group. Overall outcomes remained similar between both electrode arrays in all conditions tested, however a greater variation was measured when listening to a male speaker in noise for the SlimJ array (* p<0.006, Levene’s test).

**Figure 3:** BKB outcomes 9-12 months post activation for EAS candidates (pre-operative hearing ≤ 70 dB HL) and non-EAS candidates (pre-operative hearing >70 dB HL) based on pre-operative hearing thresholds. a) BKB scores based on 250 Hz pre-operative thresholds. b) BKB scores based on 500 Hz pre-operative thresholds. The data is plotted as back-to-back histograms, where the size of the coloured bars reflects the number of individuals with BKB scores in that % range. The number of measurements in each listening condition is listed for SlimJ (teal) and Mid-Scala (pink) electrodes, with the mean (+) and median (☐) shown for each group. Results for female speaker in noise are not shown as the sample size was too small. Differences were only detected in BKB male speaker conditions between EAS candidates (pre-operative hearing ≤ 70dB HL) and non-EAS candidates (pre-operative hearing >70 dB HL), regardless of electrode type. For 500 Hz, BKB scores when listening to a male speaker in noise were lower for non-EAS candidates with the SlimJ compared to the Mid-Scala electrode (*p<0.05, ANOVA).

**Figure 4:** Average threshold (T) and comfort (M) levels at each site for a) adult patients and b) paediatric patients who attended their 9-12 month programming appointments. All levels are shown as charge units (cu) obtained from the SoundWave programming software. * T and M levels varied across electrode contacts (1-15) for adult and paediatric patients (F>7, p <0.001, ANOVA). ** M levels in adults only showed an interaction between channel and electrode array (SlimJ/MS). ^+^ Centre 3 was not included in ANOVA, however show higher M levels.

**Supplementary Figure 3:** Average threshold (T) and comfort (M) levels at each site shown in charge (nC/phase) for a) adult patients and b) paediatric patients who attended their 9-12 month programming appointments.
